# Supplementary material for: A UHPLC-UV Method Development and Validation for Determining Kavalactones and Flavokavains in Piper methysticum (Kava)
Source: Molecules. 2019 Mar 30;24(7):1245. doi: 10.3390/molecules24071245 (PMC6479543; doi:10.3390/molecules24071245)
Supplement: Supplementary file 1 [file molecules-24-01245-s001.pdf]

TITLE: A UHPLC method Development and Validation for Determination of Kavalactones and Flavokavains in Piper methysticum (Kava).

Yijin Tang[a*] & Christine Fields[a*]

[a]Applied Food Sciences, Inc., 2500 Crosspark Road, Coralville, IA 52241

KEYWORDS: Piper methysticum (Kava), kavalactones, flavokavains, (U)HPLC-UV, Mass spectra, isomerization, single-laboratory validation, quality control

Support Figures Legend

Support Figure 1.	UHPLC chromatography of Kava standard mixture and mass spectra of six major Kavalactones and three flavokavains.
Support Figure 2.	UHPLC chromatography of Kava standard mixture and individual six major Kavalactones and three flavokavains
Support Figure 3.	Calibration curves of Kava standards of six major Kavalactones and three flavokavains.

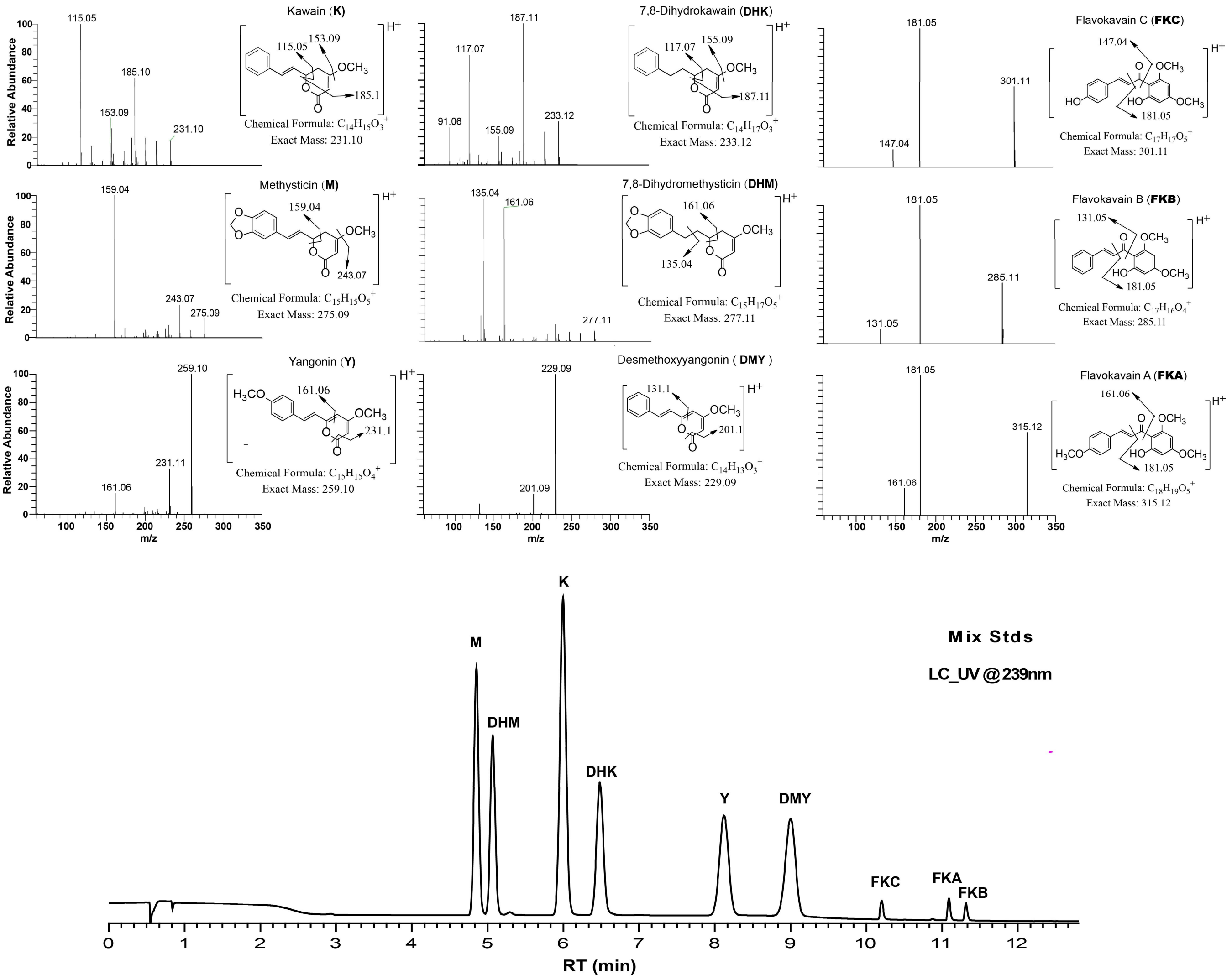

Support Figure 1

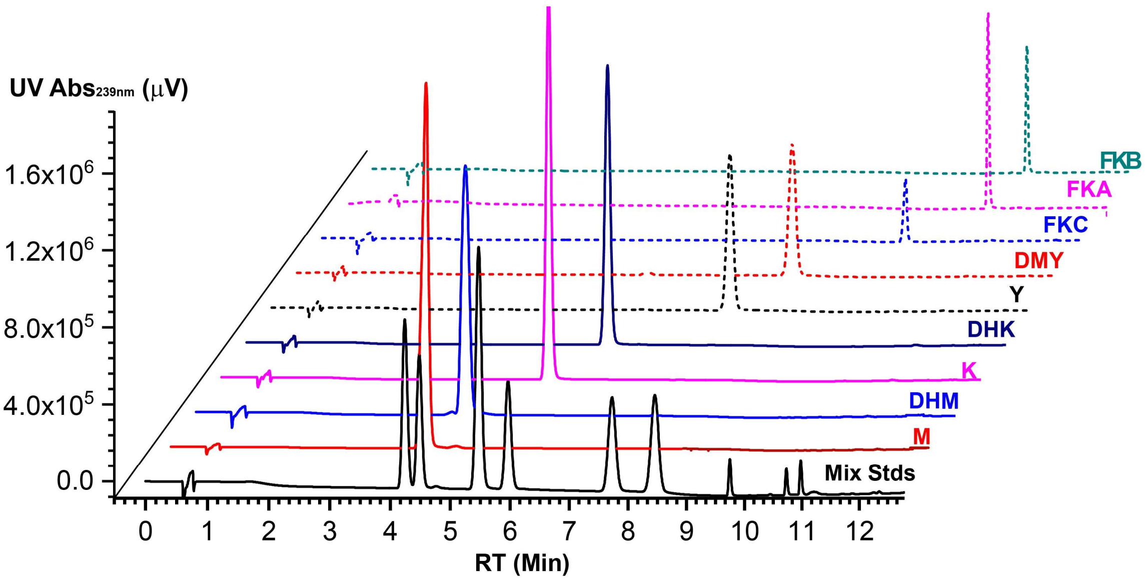

Support Figure 2

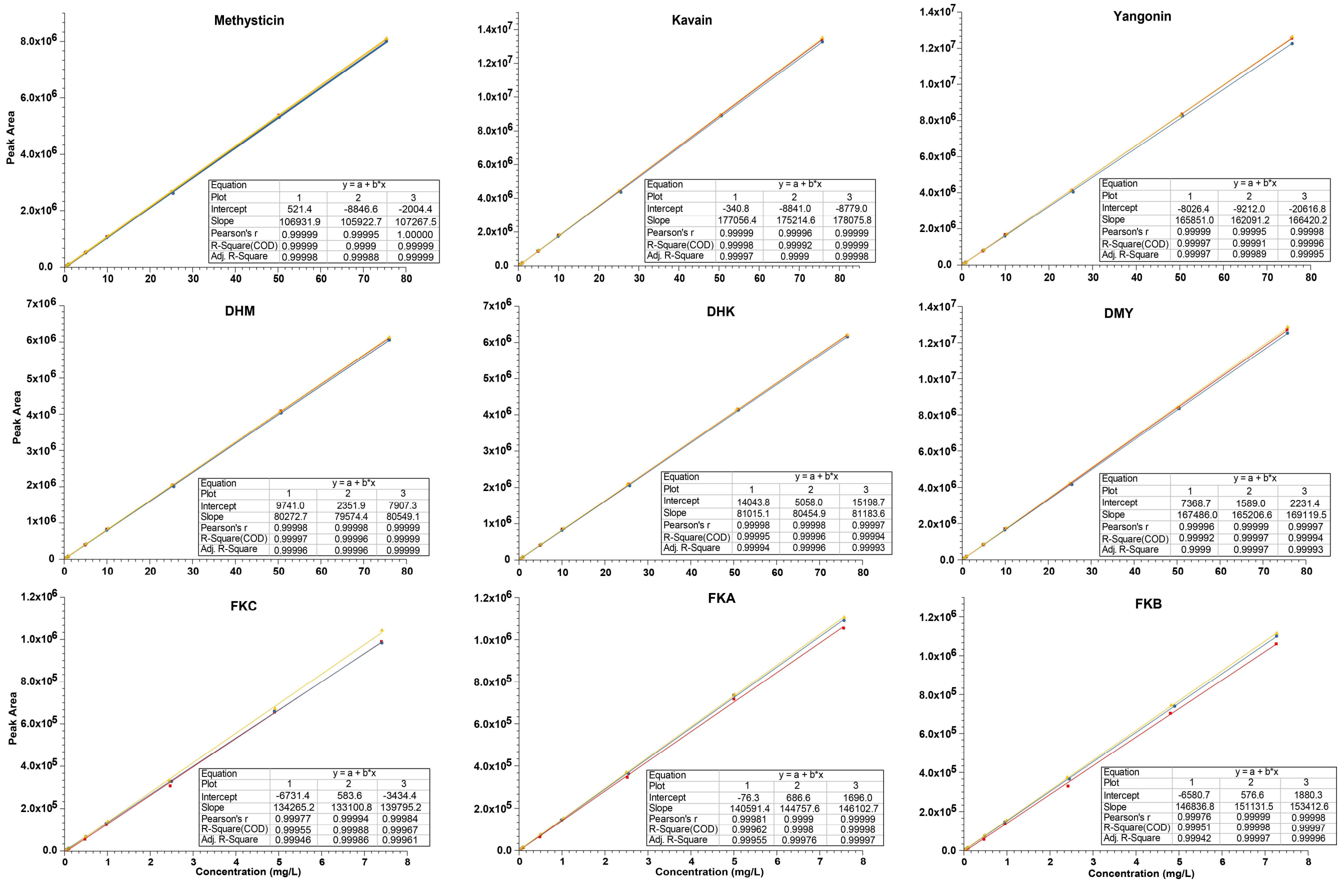

Support Figure 3
